# Long Non-Coding RNA *HOTAIR* Promotes Cell Migration and Invasion via Down-Regulation of RNA Binding Motif Protein 38 in Hepatocellular Carcinoma Cells

**DOI:** 10.3390/ijms15034060

**Published:** 2014-03-06

**Authors:** Chaofeng Ding, Shaobing Cheng, Zhe Yang, Zhen Lv, Heng Xiao, Chengli Du, Chuanhui Peng, Haiyang Xie, Lin Zhou, Jian Wu, Shusen Zheng

**Affiliations:** 1 Division of Hepatobiliary and Pancreatic Surgery, Department of Surgery, First Affiliated Hospital, School of Medicine, Zhejiang University, Hangzhou 310003, Zhejiang, China; E-Mails: dcf25@126.com (C.D.); yangzhe_0201730@163.com (Z.Y.); xiehy@zju.edu.cn (H.X.); linzhou19@163.com (L.Z.); 2 Key Laboratory of Combined Multi-Organ Transplantation, Ministry of Public Health, Hangzhou 310003, Zhejiang, China; E-Mails: showcheng@126.com (S.C.); lvzhen@zju.edu.cn (Z.L.); xiaohengdoctor@126.com (H.X.); 496722098@163.com (C.D.); clarkpch@163.com (C.P.)

**Keywords:** RNA binding motif protein 38, HOX transcript antisense RNA, hepatocellular carcinoma, long non-coding RNA

## Abstract

Long non-coding RNA *HOTAIR* exerts regulatory functions in various biological processes in cancer cells, such as proliferation, apoptosis, mobility, and invasion. We previously found that *HOX transcript antisense RNA (HOTAIR)* is a negative prognostic factor and exhibits oncogenic activity in hepatocellular carcinoma (HCC). In this study, we aimed to investigate the role and molecular mechanism of *HOTAIR* in promoting HCC cell migration and invasion. Firstly, we profiled its gene expression pattern by microarray analysis of *HOTAIR* loss in Bel-7402 HCC cell line. The results showed that 129 genes were significantly down-regulated, while 167 genes were significantly up-regulated (fold change >2, *p* < 0.05). Bioinformatics analysis indicated that RNA binding proteins were involved in this biological process. *HOTAIR* suppression using RNAi strategy with HepG2 and Bel-7402 cells increased the mRNA and protein expression levels of RNA binding motif protein 38 (RBM38). Moreover, the expression levels of RBM38 in HCC specimens were significantly lower than paired adjacent noncancerous tissues. In addition, knockdown of *HOTAIR* resulted in a decrease of cell migration and invasion, which could be specifically rescued by down-regulation of RBM38. Taken together, *HOTAIR* could promote migration and invasion of HCC cells by inhibiting RBM38, which indicated critical roles of *HOTAIR* and RBM38 in HCC progression.

## 1. Introduction

Hepatocellular carcinoma (HCC) is the second leading cause of cancer related deaths worldwide [1]. China accounts for 55% of the world’s cases each year, due to high incidence of chronic hepatitis B infection and liver cirrhosis [2]. Although great advances in surgical technique and medical care have been achieved over the last two decades, long-term survival of HCC is still low due to the high rate of recurrence and metastasis [3,4]. Moreover, the majority of HCC patients present with advanced metastatic disease at the initial diagnosis. It has been identified that the development and progression of HCC are promoted by multiple abnormal biological processes, such as gene mutations [5], epigenetic alterations [6], and dysregulation of both coding and non-coding genes [7]. For accurate diagnosis and adequate treatment of HCC, there is an urgent need to understand the precise molecular mechanisms underlying HCC pathogenesis and progression.

According to the size, non-coding RNAs (ncRNAs) are subdivided into two major classes: small ncRNAs (<200 nt) and long ncRNAs (lncRNAs, >200 nt) [8]. In recent years, microRNAs have been characterized as oncogenes or tumor suppressor genes to influence biological function of cancer cells through post-transcriptional regulation of protein expression [9]. In contrast, lncRNAs were once considered to be transcriptional noise. However, it has become increasingly clear that lncRNAs execute important functions at various levels, including X chromosomal inactivation, chromatin remodeling, and transcriptional repression [10–12].

*HOX transcript antisense RNA (HOTAIR)*, as a lncRNA, can regulate gene expression through changes in chromatin states and epigenetic modifications [13,14]. *HOTAIR*, located in the *HOXC* cluster, trimethylates histone H3 lysine-27 (H3K27me3) of the *HOXD* locus with the polycomb-repressive complex 2 (PRC2), and subsequently inhibits *HOXD* gene expression [15]. Recently, the up-regulation of *HOTAIR* was observed in several cancers, including breast cancer [14,16,17], hepatocellular carcinoma [18], colorectal cancer (CRC) [19], pancreatic cancer [20], non-small cell lung cancer (NSCLC) [21], and esophageal squamous cell carcinoma (ESCC) [22,23]. Furthermore, *HOTAIR* promoted migration and invasion of breast carcinoma cells [14], CRC cells [19], pancreatic cancer cells [20], NSCLC cells [21], and ESCC cells [22,23]. Our previous studies [18] have shown that *HOTAIR* is overexpressed in HCC and serves as an independent prognostic factor for recurrence related survival. However, the role and molecular mechanism of *HOTAIR* in promoting HCC cell migration and invasion remain to be elucidated.

In this study, we profiled its gene expression pattern by microarray analysis of *HOTAIR* loss in Bel-7402 HCC cell line. Microarray analysis revealed that the expression of QKI, CD82, and RBM38 increased in siHOTAIR groups compared with control groups, which were validated by qRT-PCR and Western blot. Moreover, the expression levels of RBM38, not QKI or CD82, were significantly lower in HCC tissues than paired adjacent noncancerous tissues. In addition, the effects of *HOTAIR* knockdown on cell migration and invasion could be specifically rescued by down-regulation of RBM38. These results suggest that *HOTAIR* promotes cell migration and invasion via suppressing RBM38, which indicated critical roles of *HOTAIR* and RBM38 in HCC progression.

## 2. Results and Discussion

### 2.1. Results

#### 2.1.1. Knockdown of *HOTAIR* Altered Global Gene Expression Patterns in HCC Cells

**Figure 1.**
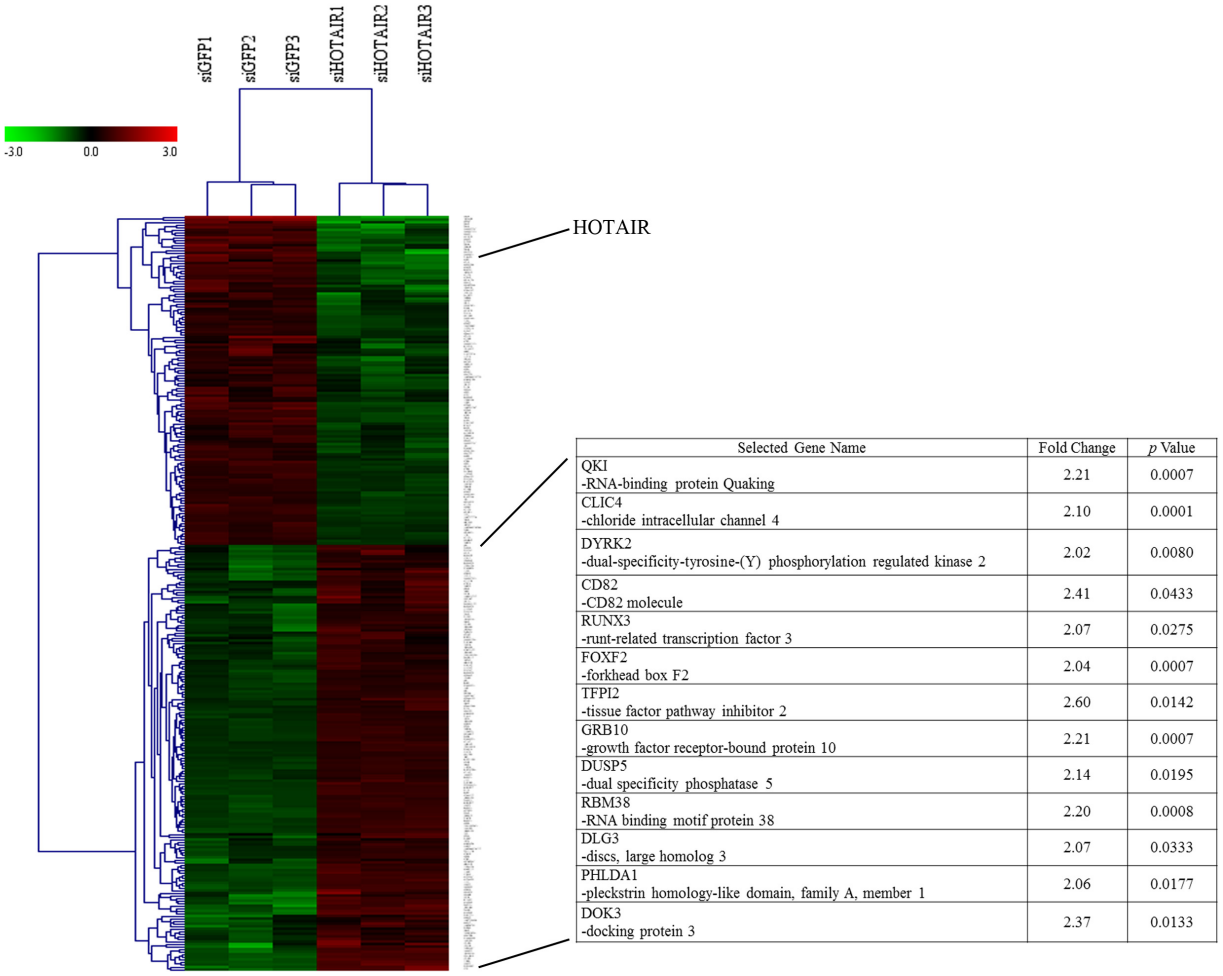
Global transcriptional changes by microarray analysis of *HOTAIR* loss in Bel-7402 HCC cell line. (**Left**), Heat map depicting transcript profiling of independent biologic replicate Bel-7402 cells with siRNA-mediated *HOTAIR* depletion (siHOTAIR) *versus* control siRNA (siGFP). Red represents upregulated genes while green represents downregulated genes; (**Right**), selected genes for further validation.

**Figure 2.**
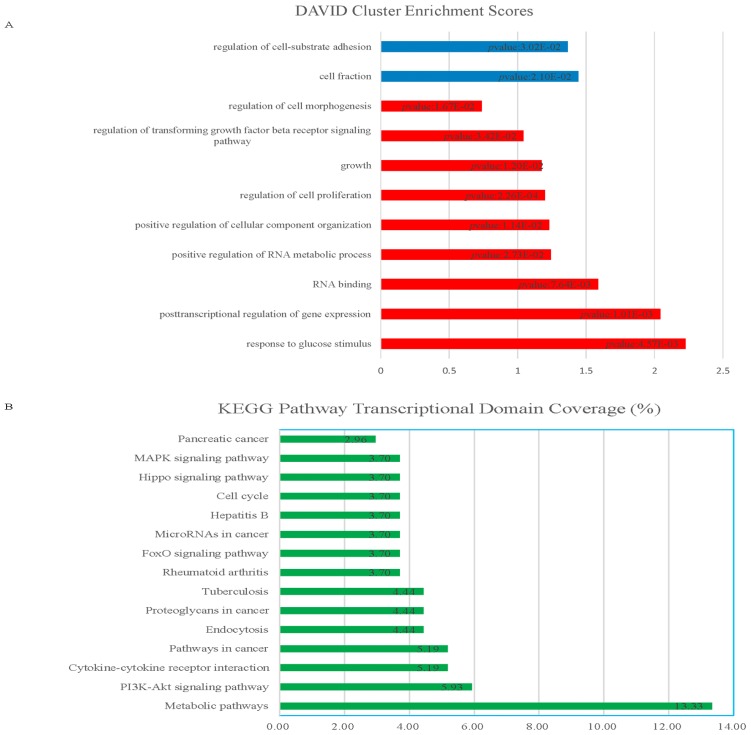

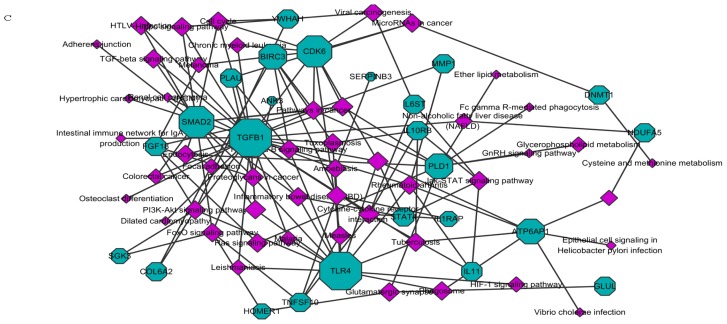
Bioinformatics analysis of *HOTAIR* loss in Bel-7402 HCC cell line. (**A**) Gene ontology analysis of *HOTAIR* knockdown microarray data using the DAVID program. Red bars represent the top hits for upregulated genes. Blue bars represent the top hits for downregulated genes. DAVID enrichment scores are represented with Benjamini-Hochberg-adjusted *p* values. All error bars in this figure are mean ± S.E.M.; (**B**) KEGG pathway analysis of *HOTAIR* knockdown microarray data. Green bars represent the percentage of transcriptional domain coverage; and (**C**) mRNA-signaling pathway integrated network based on the predicted interactions (partial).

To study the molecular mechanism of *HOTAIR* knockdown, we profiled its gene expression pattern by microarray analysis. Affymetrix u133 pluss 2.0 was applied to screen for global transcriptional changes in Bel-7402 cells 48 h after siRNA treatment. Overall, 296 genes were differentially expressed in *HOTAIR* knockdown cells (including 167 up-regulated and 129 down-regulated genes, fold change >2.0 and *p* value <0.05) (Figure 1). Gene ontology (GO) analysis showed that many differentially expressed genes are involved in biological processes relevant to cancer pathogenesis, such as metabolic process, posttranscriptional regulation of gene expression, RNA binding, cell proliferation, growth, transforming growth factor beta (TGF-β) signaling, and so on (Figure 2A). Kyoto Encyclopedia of Genes and Genomes (KEGG) pathway analysis indicated that metabolic pathways, PI3K-AKT signaling pathway, cytokine receptor interaction are top canonical pathways identified in this process (Figure 2B). Based on the predicted interactions of key molecules with their associated pathway, the mRNA-signaling pathway integrated network was built (Figure 2C and Supplementary Figure S1). Notably, TGF-β signaling pathway played an important role in this network.

Based on the results of bioinformatics analysis and the fact that overexpression of *HOTAIR* in cancer cells suppresses downstream gene expression, and increases cancer invasiveness and metastasis [14], we gave a special attention to those genes which were significantly upregulated in the microarray data and had been reported as potential tumor suppressors. After a screening of 167 genes, 13 genes of them met the above requirements. These genes were QKI, CLIC4, DYRK2, CD82, RUNX3, FOXF2, TFPI2, GRB10, DUSP5, RBM38, DLG3, PHLDA1, and DOK3 (Figure 1 right).

#### 2.1.2. Upregulation of QKI, CD82, and RBM38 Were Validated after Knockdown of *HOTAIR*

**Figure 3.**
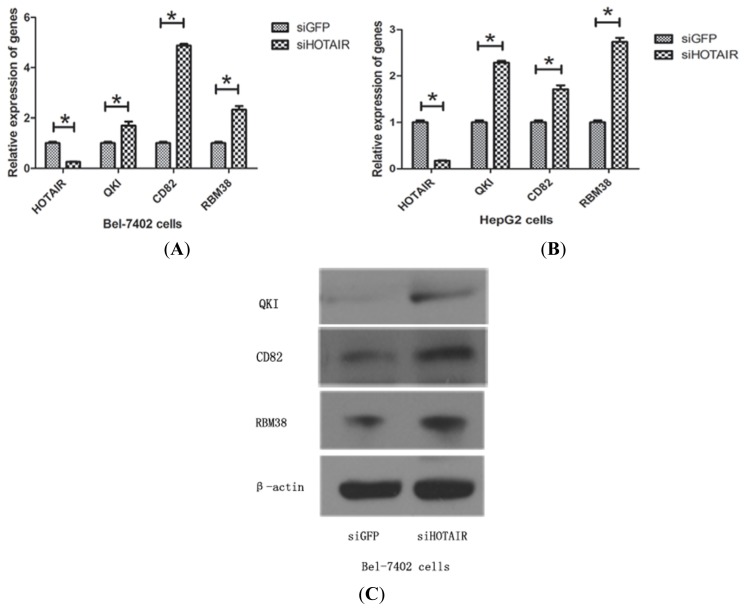
Upregulation of QKI, CD82, and RBM38 were validated after knockdown of *HOTAIR*. (**A**) Bel-7402 cells; (**B**) HepG2 cells were transiently transfected with *HOTAIR* siRNA for 48 h to detect QKI, CD82, and RBM38 expression by qRT-PCR compared with the siGFP group (* indicated *p* < 0.05); and (**C**) showed the protein level of QKI, CD82, and RBM38 after *HOTAIR* knockdown in Bel-7402 cells by Western blot. All experiments were performed in triplicates.

Thirteen upregulated genes were selected for further analysis. Previous studies showed that all of these genes had potential tumor suppressor roles in tumor development and progression. *HOTAIR* siRNA treatment was introduced to Bel-7402 and HepG2 cells. Then mRNA levels of these genes were assessed after 48 h transfection. Finally, we found three genes (QKI, CD82, and RBM38) significantly upregulated after *HOTAIR* knockdown in Bel-7402 cells (Figure 3A) and HepG2 cells (Figure 3B). Moreover, the protein levels of QKI, CD82, and RBM38 were increased in siHOTAIR group compared with siGFP group by Western blot (Figure 3C). These results suggest that downregulation of *HOTAIR* can increase QKI, CD82, and RBM38 expression both on mRNA and protein levels.

#### 2.1.3. QKI, CD82, and RBM38 Knockdown by the Corresponding siRNAs Did not Alter Cell Proliferation

**Figure 4.**
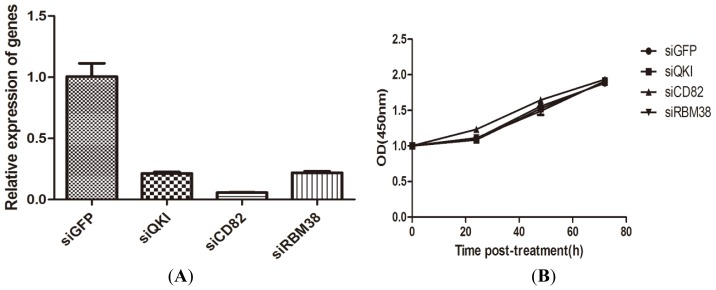
Effects of QKI, CD82, and RBM38 knockdown on cell proliferation. (**A**) effective silencing of QKI, CD82, and RBM38 expression in Bel-7402 cells after 48 h siRNA treatment according to qRT-PCR analysis; and (**B**) Viable cell numbers were detected by CCK-8 assay after transferred to 96-wells plates for 24, 48, and 72 h.

To selectively suppress the expression of QKI, CD82, and RBM38, we used the gene-specific siRNAs (QKI-siRNA, CD82-siRNA and RBM38-siRNA) against QKI, CD82, and RBM38 in Bel-7402 cells. qRT-PCR results showed that QKI, CD82 and RBM38 mRNA levels were significantly reduced by the corresponding siRNAs treatment compared with siGFP groups (Figure 4A). Next we analyzed the effects of silencing of QKI, CD82, and RBM38 on HCC cell proliferation. CCK-8 assays showed that knockdown of these genes did not alter proliferation of Bel-7402 cells compared with siGFP groups (Figure 4B). These results indicated that these genes have little effect on HCC cell proliferation.

#### 2.1.4. The Expression Levels of RBM38 Were Decreased in HCC Samples

**Figure 5.**
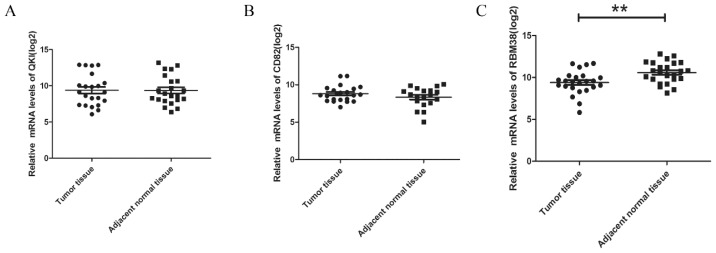
The expression levels of RBM38 were downregulated in HCC resection specimens. The expression levels of QKI (**A**); CD82 (**B**) and RBM38 (**C**) in HCC and paired adjacent noncancerous tissues were measured by qRT-PCR and normalized to GAPDH. The significant differences between samples were analyzed using the Student’s *t* test. (log 2, ** *p* < 0.01).

We detected the mRNA levels of QKI, CD82, and RBM38 in 53 pairs of HCC resection specimens by qRT-PCR. Each HCC pair contained the cancer and the corresponding adjacent normal tissues. No significant change for QKI and CD82 were observed in 53 paired HCC samples (Figure 5A,B). However, the relative expression levels of RBM38 were significantly downregulated in HCC tissues compared with paired adjacent noncancerous tissues (*p* = 0.0043) (Figure 5C).

To explore whether RBM38 could be an important biomarker for HCC patients, we evaluated the relationship between RBM38 expression and clinicopathological parameters. Thirty-nine of these cases (73.6%) exhibited lower expression levels of RBM38. The cutoff value for high and low expression of RBM38 was the mean value of RBM38 mRNA level. Reduced expression of RBM38 negatively correlated with serum AFP level (*p* = 0.017). There was no significant correlation between RBM38 expression and other clinicopathological features (Table 1).

#### 2.1.5. RBM38 Knockdown Promoted Migration and Invasion of HCC Cells

**Figure 6.**
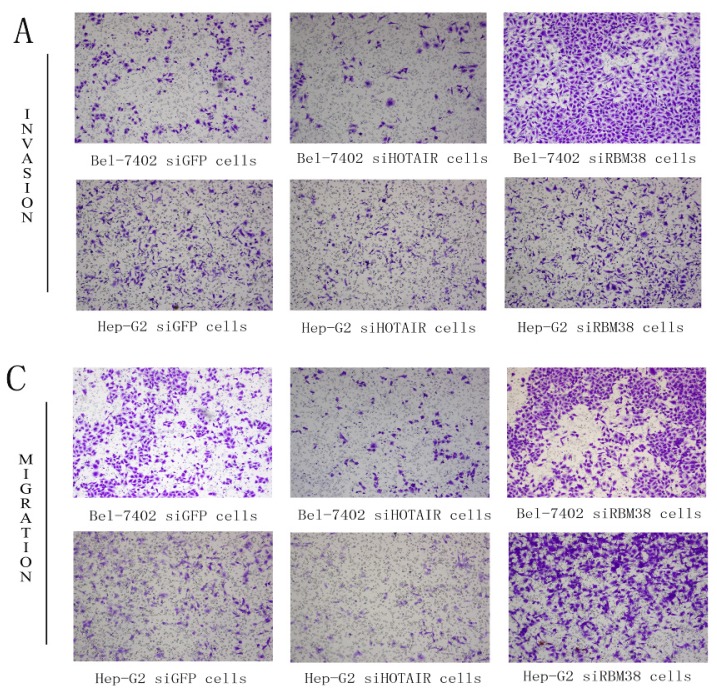

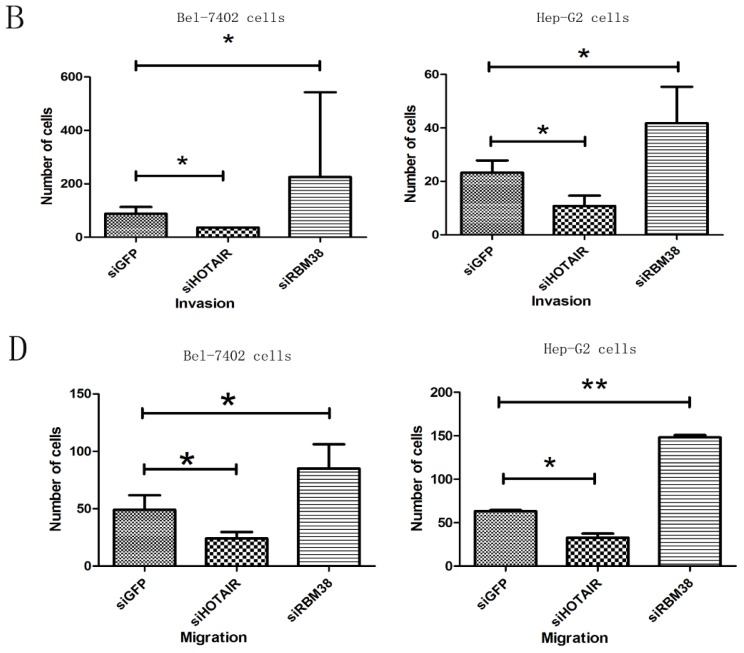
RBM38 inhibited the invasion and migration of HCC cells. Representative images of invasion (**A**) and migration (**C**) of Bel-7402 cells and HepG2 cells transfected with siGFP, siHOTAIR and siRBM38; The number of invaded cells was measured and represented in (**B**) and (**D**), respectively. The representative invasion images are shown at 100× magnification.* *p* < 0.05, ** *p* < 0.01.

To examine the effect of RBM38 knockdown in liver cancer cells on migration and invasion, we developed a transwell assay with Bel-7402 and HepG2 cells. The siRBM38-transfected cells significantly increased invasion and migration ability compared with the siGFP group in Bel-7402 cells and HepG2 cells (Figure 6A–D). These findings showed that RBM38 could inhibit cell migration and invasion.

#### 2.1.6. *HOTAIR* Is Functionally Related to RBM38

**Figure 7.**
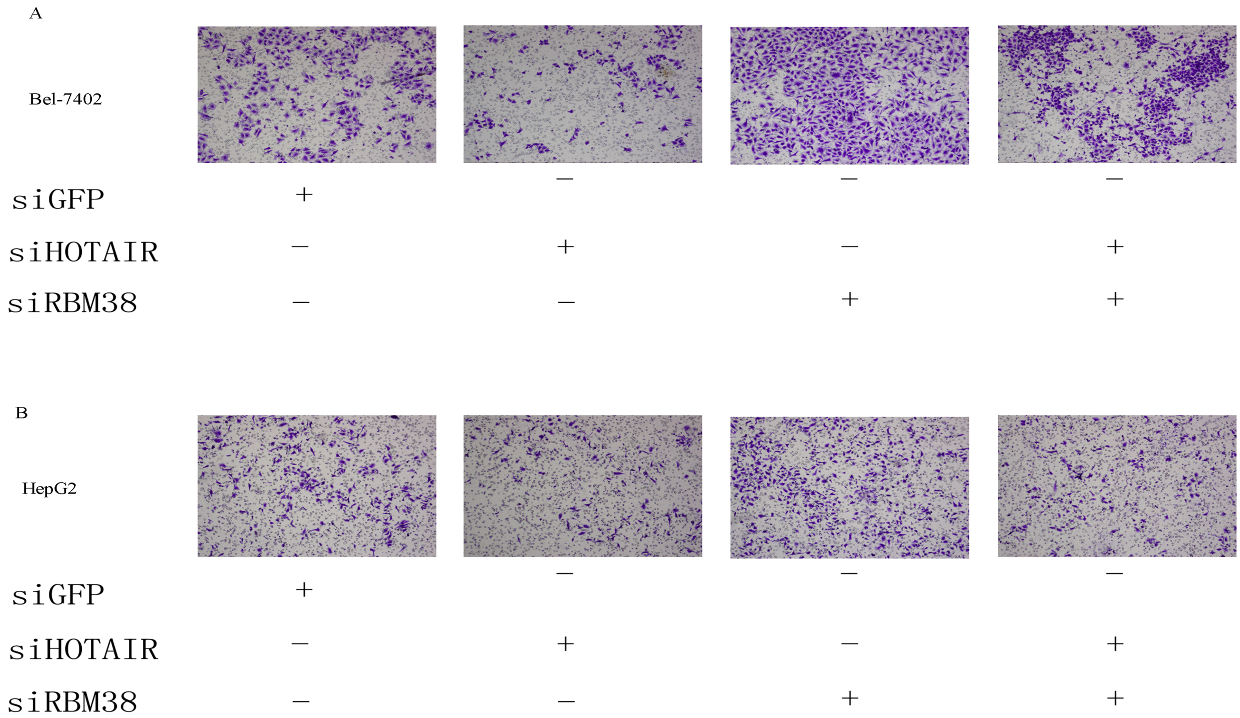
Knockdown of RBM38 restored the *HOTAIR* siRNA-mediated inhibition of invasion in Bel-7402 cells (**A**) and HepG2 cells (**B**). The number of invaded cells was measured. The representative invasion images are shown at 100× magnification.

Although *HOTAIR* was shown to negatively regulate RBM38, it is unknown whether a direct or indirect functional relevance to cancer cell invasion exists. In order to determine the relationship between *HOTAIR* and RBM38, HCC cells were co-transfected with siHOTAIR and siRBM38 and compared with those only transfected with siHOTAIR, siRBM38 or siGFP. The invasion ability of HCC cells transfected with siHOTAIR were decreased compared with siGFP group. On the contrary, the cells transfected with siRBM38 had increased motility. Furthermore, RBM38 knockdown could restore siHOTAIR-mediated inhibition of motility in HCC cells (Figure 7A,B). Thus, these findings indicated that the effect of *HOTAIR* knockdown on cell motility could be specifically rescued by downregulation of RBM38, thereby, confirming the functional relevance between *HOTAIR* and RBM38.

### 2.2. Discussion

Long noncoding RNAs represent a novel class of noncoding RNAs that are longer than 200 nucleotides without protein-coding potential [8]. Recently, lncRNAs were found to be dysregulated and involved in various cancer biological processes, such as proliferation, apoptosis, mobility, and invasion [10,24]. But the underlying mechanism remains uncertain. Therefore, understanding the role and precise molecular mechanism of lncRNAs in cancer development and progression are urgently needed.

In our previous study [18], *HOTAIR* was increased in HCC tissues compared with paired noncancerous tissues. Higher *HOTAIR* expression level was an independent prognostic marker for HCC recurrence and shorter survival. However, the role and molecular mechanism of *HOTAIR* in promoting HCC cell migration and invasion are largely limited.

In this study, we profiled its gene expression pattern by microarray analysis of *HOTAIR* loss in Bel-7402 HCC cell line. We found that 296 genes were differentially expressed in *HOTAIR* knockdown cells, including 167 up-regulated and 129 down-regulated. GO and pathway analysis indicated the differentially expression genes were significantly enriched in processes, such as cell metabolism, cell proliferation, growth, cell adhesion, and so on, providing important clues for understanding molecular pathogenesis of HCC. Furthermore, several RNA binding proteins involved in this biological process caught our attention, such as QKI and RBM38. Microarray analysis revealed that QKI and RBM38 expression increased in siHOTAIR groups compared with control groups, which were selected and subsequently validated by qRT-PCR and Western blot. These findings suggest RNA binding proteins were downstream molecules repressed by *HOTAIR*. Interestingly, we found that TGF-β signaling pathway might play an important role in the network according to the integrated KEGG pathway analysis. Our findings are in agreement with Alves’s reports [25], in which *HOTAIR* acted as a regulator in TGF-β inducing epithelial-to-mesenchymal transition process.

Additionally, *in vivo* experiments have shown that the expression levels of RBM38, not QKI, were decreased in human HCC tissues compared with paired noncancerous tissues. Moreover, the expression of RBM38 negatively correlated with serum AFP level. These findings suggest that RBM38 may serve as a potential tumor suppressor in HCC. Consistently, Leveille *et al.* found the reduced expression of RBM38 in cohorts of human breast cancer [26]. RBM38 has also been described as a target of p53, and induces cell cycle arrest in G1 by stabilizing the CDK inhibitor p21 [27].

To confirm the functional relevance between *HOTAIR* and RBM38, we compared the cell motility among HCC cells with siRBM38, siHOTAIR, and co-transfected with siHOTAIR and siRBM38. Silencing of RBM38 could restore cell motility, while knockdown of *HOTAIR* significantly reduced cell motility. Furthermore, HCC cells co-transfected with siHOTAIR and siRBM38 could rescue cell motility inhibition by *HOTAIR* knockdown. These findings suggest that *HOTAIR* knockdown could reduce cell motility probably by increasing RBM38, which indicate RBM38 as a repressed target of *HOTAIR*.

Many lncRNAs have been documented to promote tumor progression and metastasis via several different pathways: transcriptional silencing through recruitment of epigenetic complex to specific loci [28]; posttranscriptional gene regulation [29,30]; and decoys for miRNAs and splicing factors [31,32]. Gupta *et al.* reported that *HOTAIR* could recruit PRC2 to *HOXD* locus, trimethylated H3K27me3, increasing breast cancer invasiveness and metastasis [14]. Similarly, Ge *et al.* reported that *HOTAIR* directly promoted H3K27me3 in the promoter region of WIF-1, a key regulator in Wnt/β-catenin signaling pathway, and thereby decreased its expression to promote the ESCC cell invasion [22]. Our studies focused on the regulatory roles of RBM38 in HCC cell migration and invasion, and its functional relevance with *HOTAIR*. However, further studies are needed to confirm whether *HOTAIR* promoted H3K27me3 in the promoter region of RBM38 to suppress its expression.

In summary, we profiled the gene expression pattern by microarray analysis of *HOTAIR* loss in Bel-7402 HCC cell line. The expression levels of QKI, CD82, and RBM38 were increased after *HOTAIR* knockdown in microarray data, which were further validated by qRT-PCR and Western blot. Moreover, the expression levels of RBM38, not QKI or CD82, were significantly lower in HCC tissues than paired adjacent noncancerous tissues. In addition, knockdown of *HOTAIR* resulted in a decrease of cell migration and invasion, which could be specifically rescued by down-regulation of RBM38. Taken together, *HOTAIR* could promote migration and invasion of HCC cells by inhibiting RBM38, which indicated critical roles of *HOTAIR* and RBM38 in HCC progression.

## 3. Experimental Section

### 3.1. Patient Samples

Fifty-three cases of patients who underwent surgery between August 2007 and May 2011, in the First Affiliated Hospital, Zhejiang University School of Medicine were enrolled in this study. This study was approved by the Ethics Committee of the hospital and was performed after obtaining written informed consent of patients. Specimens were obtained immediately after surgical resection and stored at −80 °C for further analysis. There were 44 men and 9 women, ranging in age from 29 to 78 years, with an average age of 51.6 years.

### 3.2. Materials

Liver cancer cell lines HepG2 and Bel-7402 were purchased from Shanghai Institute of Cell Biology (Shanghai, China). All of the cell lines were maintained in the recommended culture condition and incubated at 37 °C in a humidified environment containing 5% CO_2_.

### 3.3. Microarray Experiments and Data Analysis

Briefly, Bel-7402 cells were transfected with *HOTAIR* siRNA or siGFP in triplicate. Forty-eight hours after transfection, total RNA was extracted using the Trizol reagent (Invitrogen, Carlsbad, CA, USA) and integrity assessed with an Agilent Bioanalyzer 2100 (Agilent Technologies, Palo Alto, CA, USA). The RNA was then labeled and hybridized to U133 Plus 2.0 arrays (Affymetrix, Santa Clara, CA, USA) according to the manufacturer’s instructions. Raw microarray data were normalized using Affymetrix Expression Console Software Version 4.0 (Affymetrix, Santa Clara, CA, USA) and Log 2-transformed. The transformed data of two groups were compared using Welch’s *t*-test. The threshold set for up- and down-regulated genes was a fold change >2.0 and a *p* value <0.05. GO analysis and KEGG analysis were applied to determine the roles of these differentially expressed mRNAs. Finally, Hierarchical clustering was performed to display the distinguishable genes’ expression pattern among samples.

### 3.4. RNA Isolation and Real-Time Quantitative Reverse-Transcription Polymerase Chain Reaction (qRT-PCR)

Total RNA from cells was extracted using Trizol reagent (Invitrogen) and cDNA was synthesized with M-MLV Reverse Transcriptase (Promega, Madison, WI, USA). The primers for qRT-PCR were synthesized from Genepharma Inc. (Shanghai, China). QRT-PCR reactions were performed by ABI7500 Fast (Applied Biosystems, Carlsbad, CA, USA) with the SYBR Premix Dimmer Eraser kits (TaKaRa, Dalian, China). *Glyceraldehyde 3-phosphate dehydrogenase (GAPDH)* was used as an internal control to normalize target mRNA levels. The relative expression was calculated by the 2^−ΔΔ^*^C^*^t^ method. The nucleotide sequences of the primers were as follows:

GAPDH-F: ATGGGGAAGGTGAAGGTCG
GAPDH-R: GGGGTCATTGATGGCAACAATA
CD82-F: CTGCAGGATGCCTGGGACTA
CD82-R: CTCAGCGTTGTCTGTCCAGTTGTA
QKI-F: GATGCAGCTGATGAACGACAAG
QKI-R: CAGCATCAGGCAATTCTGCAC
RBM38-F: GATACTGCCGCCACCAAGA
RBM38-R: CGAGCCGGCAGACACTTTATTATAC

### 3.5. Small Interfering RNA Transfection

Untreated cells were plated at 3 × 10^5^/well in 2 mL of medium for 24 h before transfection. HepG2 and Bel-7402 cells were transfected with 50 nM siRNAs targeting genes or siGFP (GenePharma, Shanghai, China) using Lipofectamine2000 transfection reagent (Invitrogen, Carlsbad, CA, USA) according to the manufacturer’s instructions, The mRNA of HOTAIR, QKI, RBM38 and CD82 was harvest after siRNA transfection for 48 h. The siRNA sequences were listed as follows:

siQKI: CCUUGAGUAUCCUAUUGAACCUAGU
siRBM38: GACACCACGUUCACCAAGA
siCD82: UAUUUGGUGACUUUGAUACAGGCUG
siGFP: CUACAACAGCCACAACGUCTT
siHOTAIR: GAACGGGAGUACAGAGAGAUU

### 3.6. Cell Proliferation Assay

Cell proliferation was detected using a cell counting kit-8 (Dojindo Laboratories, Kumamoto, Japan) according to the manufacturer’s instructions after transfecting with siRNA in 6-well plates, and were transferred to 96-well plates with 3000 cells per well. After 24, 48, and 72 h incubation in 96-well plates, the relative numbers of viable cells were represented by the absorbance optical density at 450 nm using a microplate reader (Elx800; BioTek, Winooski, VT, USA).

### 3.7. Cell Migration and Invasion Assay

After being washed with PBS, HepG2 or Bel-7402 cells were detached with trypsin, and resuspended in serum free medium. Then, 200 μL of cell suspensions (2.5 × 10^5^ cells/mL) was added to the upper chamber with non-coated membrane (24-well insert; 8-mm pore size; Millipore, Billerica, MA, USA) or coated with Matrigel (BD Bioscience, Bedford, MA, USA) for the transwell migration or invasion assays, respectively. Culture medium containing 10% FBS was added to the bottom wells of the chambers. The cells were incubated for 24 h (migration assay) or 48 h (invasion assay). After 24 or 48 h, the cells that had still stayed in the upper face of the filters were removed using cotton swabs, and the cells that had migrated to the lower face of the filters were fixed with 100% methanol and stained with 0.2% crystal violet and counted. The mean of triplicate assays for each experimental condition was used.

### 3.8. Protein Extraction and Western Blot Analysis

After incubation, cells were collected and lysed in lysis buffer (50 mL Tris (pH 7.4), 150 mL sodium chloride, 1% Triton X-100, 1% sodium deoxycholate, 0.1% sodium dodecyl sulfate, sodium orthovanadate, sodium fluoride, EDTA, and leupeptin supplemented with 1 mm phenylmethanesulfonyl fluoride) at 4 °C. The lysate was vibrated three times for 10 min each and sonicated, followed by centrifugation at 13,000 rpm for 20 min at 4 °C. Soluble proteins were collected and stored at −80 °C. The protein concentrations were determined using a BCA Protein Assay Kit (Pierce, Rockford, IL, USA) by Bradford assay (Bio-Rad, Hercules, CA, USA). 25~30 μg of total protein was applied for detection of QKI, CD82, and RBM38 protein. After denaturation of proteins by heating at 70 °C for 10 min, the proteins were separated by gel electrophoresis using 12% SDS-PAGE, and blotted onto polyvinylidene fluoride membrane (Millipore, Billerica, MA, USA). Then, the membrane was blocked with 5% nonfat milk in Tris-buffered saline with 0.05% Tween-20 (TBST) and incubated overnight with the following primary antibodies: rabbit anti-QKI (Epitomics, Inc., Burlingame, CA, USA), rabbit anti-CD82 (Epitomics, Inc., Burlingame, CA, USA); rabbit anti-RBM38 (Abcam, Cambridge, UK); and mouse anti-β-actin (Sigma-Aldrich, St. Louis, MO, USA). After incubation with horseradish peroxidase (HRP)-conjugated secondary antibody (Jackson Immuno Research, West Grove, PA, USA), antibody binding was visualized with SuperSignal West Pico Chemiluminescent Substrate (Pierce, Billerica, MA, USA). β-actin was tested as a control.

### 3.9. Statistical Analysis

The association between RBM38 expression and clinicopathological variables was assessed by two-sided Pearson chi-square test. All statistical analysis was performed using SPSS 16.0 (Chicago, IL, USA) and GraphPad Prism 5.0 (GraphPad Software Inc., San Diego, CA, USA). The experimental data were expressed as median with range or mean ± SD where applicable. Differences between groups were determined by Student’s *t* test, and *p* < 0.05 was set to be statistically significant.

## 4. Conclusions

Our findings suggest that *HOTAIR* could promote migration and invasion of HCC cells by inhibiting RBM38, which indicated critical roles of *HOTAIR* and RBM38 in HCC progression.

## Supplementary Information



## Figures and Tables

**Table 1.** Relationship between RBM38 expression and clinicopathological features of 53 hepatocellular carcinoma (HCC) patients.

Variables	*N* = 53	RBM38 expression		*χ^2^*	*p* value
				
		high	low		
**Age (years)**					

≤50	21	6 (11.3%)	15 (28.3%)	0.083	0.773
>50	32	8 (15.1%)	24 (45.3%)		

**Gender**					

Male	44	13 (24.5%)	31 (58.5%)	1.306	0.253
Female	9	1 (1.9%)	8 (15.1%)		

**Size of tumor (cm)**					

≤5	32	7 (13.2%)	25 (47.2%)	0.856	0.355
>5	21	7 (13.2%)	14 (26.4%)		

**Number of tumor**					

Single	29	8 (15.1%)	21 (39.6%)	0.045	0.832
Multiple	24	6 (11.3%)	18 (33.9%)		

**Vascular invasion**					

Negative	41	11 (20.8%)	30 (56.6%)	0.016	0.899
Positive	12	3 (5.7%)	9 (17.0%)		

**Pathological Grading**					

Well/Moderately	25	5 (9.4%)	20 (37.7%)	1.002	0.317
Poorly	28	9 (17.0%)	19 (35.8%)		

**AFP (ng/mL)**					

≤400	20	9 (17.0%)	11 (20.8%)	5.708	0.017
>400	33	5 (9.4%)	28 (52.8%)		

**TBIL (μmol/L)**					

≤20	33	10 (18.9%)	23 (43.4%)	0.680	0.410
>20	20	4 (7.5%)	16 (30.2%)		

AFP, alpha-fetoprotein; TBIL, total bilirubin; Statistical analyses were performed with Pearson’s chi-square test.
