# Supplementary material for: Long Non-Coding RNA HOTAIR Promotes Cell Migration and Invasion via Down-Regulation of RNA Binding Motif Protein 38 in Hepatocellular Carcinoma Cells
Source: Int J Mol Sci. 2014 Mar 6;15(3):4060–76. doi: 10.3390/ijms15034060 (PMC3975384; doi:10.3390/ijms15034060)

Supplementary Information

Figure S1. mRNA-signaling pathway integrated network based on the predicted interactions.

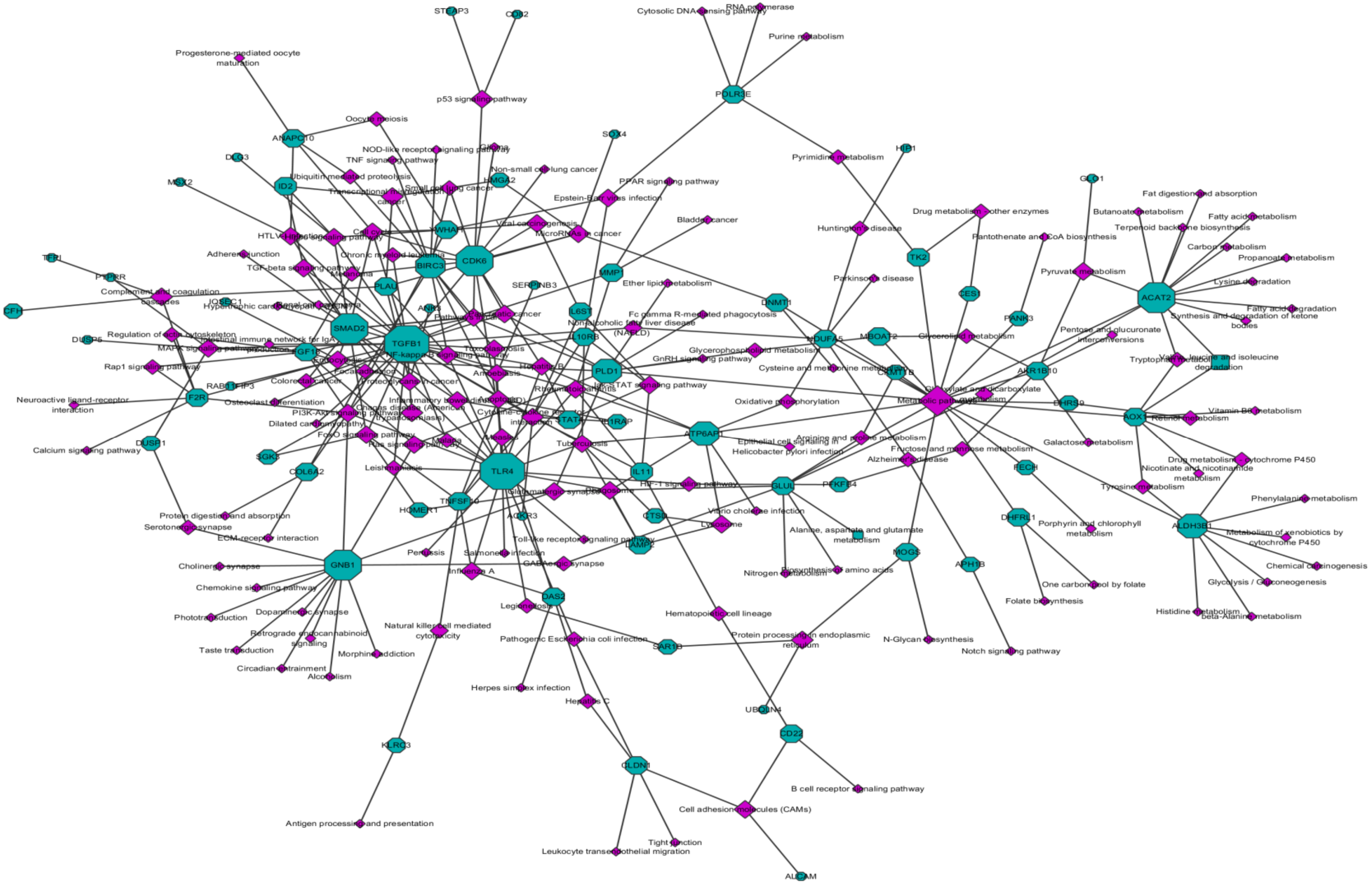

Supplement: Supplementary file 1 [file ijms-15-04060-s001.pdf]
